# Cardiovascular Risk Factors Evaluated Using the Framingham Method in a University Community Setting

**DOI:** 10.3390/biomedicines13051017

**Published:** 2025-04-23

**Authors:** Angela Mendoza, Sylvia Hidalgo, Luis Oviedo

**Affiliations:** Facultad de Ciencias de la Salud, Universidad Católica de Santiago de Guayaquil, Guayaquil 090615, Ecuador

**Keywords:** cardiovascular risk factors, Framingham method, university community

## Abstract

**Background/Objectives**: Due to their work and lifestyle, many members of the university faculty, as well as administrative staff and students, are at risk of developing health problems. These risk factors can be assessed to find out their contribution to developing heart disease. The main objective of this study is to determine cardiovascular risk factors in a university setting based on the Framingham method. **Methods**: A quantitative approach, cross-sectional design, and an observational descriptive level were used in the study. The sample was made up of 85 members of the university community, and an adapted Framingham scale was used for data collection. **Results**: The results obtained show high percentages of obesity and overweight, especially in the administrative staff, with 55% and 35%, respectively, and in teachers, with 23% and 54%, respectively. Women are at higher risk of cardiovascular events and diabetes mellitus as measured by abdominal circumference (53%), with administrative staff (65%) being the most at risk, followed by teachers (50%). In addition, the community is at risk of developing metabolic disorders (61%) as determined by the insulin secretion coefficient. HDL in administrative staff was also moderately abnormal (45%). **Conclusions**: Significant cardiovascular risks are found in the university community and an intervention based on a program to develop healthy behaviors is suggested.

## 1. Introduction

The Framingham method is a cardiovascular risk assessment tool used to predict the likelihood of a person developing cardiovascular disease. It was developed in the town of Framingham, Massachusetts, in the 1940s and has been revised and updated several times since. It uses a number of risk factors to predict an individual’s risk to develop heart disease. These factors include age, gender, blood pressure, cholesterol, blood glucose, smoking, body mass index, and family history of heart disease. Each of these factors is assessed to determine the overall risk of heart disease. The total score is used to predict a person’s likelihood of developing heart disease within the next 10 years [1].

University faculty, as well as administrative staff and students, may also be exposed to health risk factors due to their work and lifestyle. In the case of professors, the main risk factors include stress due to workload, student demands, and the pressure to publish books and articles as well as to conduct research. Also, a sedentary lifestyle is common because of long hours at a desk in front of a computer, which contributes to a lack of physical activity and increases the risk of chronic diseases; poor posture leads to an increased risk of back pain and other related problems; lack of sleep and unhealthy eating increase the risk for obesity and metabolic diseases. It is necessary to identify risk factors to avoid complications [2]. In the case of administrative staff, they experience high levels of stress and also lead a sedentary lifestyle, have poor posture and make bad dietary choices. In the case of students, they lack sleep, tend to eat unhealthy foods, exhibit dangerous habits and get stressed out due to class overload.

The incidence of cardiovascular diseases, such as ischemic and hypertensive heart disease and diabetic cardiomyopathy, is increasing at an accelerated pace worldwide, which is why they have been declared public health problems. Among the leading causes of death are: systemic arterial hypertension, diabetes mellitus, and dyslipidemia. They occur in large numbers and represent cerebrovascular and cardiovascular diseases [3].

Some of the most important cardiovascular risk factors that are taken into consideration in the Framingham scale are: age, gender, genetics, sedentary lifestyle, smoking, hypertension, diabetes, obesity, and dyslipidemia (LDL cholesterol and triglycerides).

When presenting with these risk factors, one increases the chances of experiencing acute myocardial infarction, sudden death, ischemic cerebrovascular events (temporary or definitive), and hemorrhagic cerebrovascular events.

The evaluation and stratification of cardiovascular risk factors is essential for early identification of greater predisposition for developing heart disease. The Framingham method has been widely used to estimate cardiovascular risk in various populations [3]. Cardiovascular risk factors are defined as biological characteristics or lifestyle habits that increase the probability of suffering from fatal or non-fatal cardiovascular disease. Some of the risk factors include: smoking, high blood pressure, hypercholesterolemia, gender, obesity, unhealthy diet, lack of physical exercise, ethnicity, diabetes mellitus, and having a family history of cardiovascular diseases [4].

According to the World Health Organization (WHO), heart disease and stroke are the main causes of death for more than 17 million people each year. This represents approximately one third of deaths worldwide, and that number will increase to 24 million in the year 2030 [5]. Cardiovascular risk factors (CVRF) are considered a public health problem, because they are closely related to unhealthy habits of individuals [6].

At the top of the list is leading a sedentary lifestyle, which is considered a predisposing factor associated with being overweight or obese, having hyperglycemia, and hypercholesterolemia [7]. More than 90% of myocardial infarctions are explained by classic CVRF; these are also the most important factors in the development of atherosclerosis. Many other risk factors have been identified; however, they play a rather minor role in atherosclerosis. The early detection and management of CVRF contributes favorably to a lower incidence of both coronary and cerebral events, hence the importance of preventive measures and of knowledge of the risk of developing an illness in the next 5 to 10 years [8].

Heart attacks are usually caused by a combination of factors. The first study to establish risk factors associated with cardiovascular diseases was the Framingham Heart Study, which classified them into two broad categories: non-modifiable and modifiable risk factors. The former includes age, gender, ethnicity, and family history of coronary heart disease. The latter includes high blood pressure, dyslipidemia, diabetes mellitus, obesity, smoking, lifestyle (diet and sedentary lifestyle), and stress [9].

The importance of lifestyle changes in patients with cardiovascular risk factors is high. Losing 10 kg in weight represents a 10 to 20 mmHg decrease in systolic pressure and a 5 to 10 mmHg decrease in diastolic pressure. These estimates are excellent, considering that the 10 mmHg reduction increases quality of life and extends life expectancy by up to approximately 5 years. Decreasing salt intake from 10 g to 5 g reduces blood pressure by approximately 5 to 10 mmHg. Practicing exercise such as walking, swimming, and dancing for 45 to 60 min improves cardiac inotropism and chronotropism and reduces blood pressure by 10 to 20 mmHg. Increasing the intake of potassium with foods such as bananas, tomatoes, or coconuts reduces blood pressure by approximately between 5 and 10 mmHg. Reducing the burden of work stress helps reduce high blood pressure.

## 2. Materials and Methods

A quantitative approach, cross-sectional design, and an observational descriptive level was used in the study. The sample was made up of 95 participants; however, 11 of them could not be used due to lack of information, giving us 84 participants of members of the university (teachers, laboratory assistants, security personnel, administrative staff, students from the different colleges) who voluntarily indicated they wished to participate and were asked to sign a consent form. They were asked to fill out a questionnaire that included information such as age, gender, ethnicity, and occupation. An adapted Framingham scale was used for data collection. The smoking habits information is not available as the university community is a smoking free zone and the participants would be inclined not to give accurate answers for fear of being sanctioned. Then, blood pressure was taken using digital equipment, weight and height were measured so that the Body Mass Index could be calculated, and abdominal circumference was measured in centimeters. After this, coefficient of insulin secretion, total cholesterol, triglycerides, high-density lipoproteins (HDL-C), and low-density lipoproteins (LDL-C) measurements were taken by means of a sensitive measuring device called Non-Invasive Quantum Resonance Magnetic Analyzer, which analyzes the health of internal organs by measuring the body’s energy frequency using a magnetic field sensor installed in a handle and an electrode sensor. This device works on the principle of magnetic resonance by creating a low magnetic field that interacts with the body’s magnetic field, capturing and analyzing the changes. This method is claimed to determine the patient’s condition with an accuracy of 85% [10]. No previous preparation is required. Each participant is asked to stand up straight, hold the handles of the device by stretching their arms out horizontally, and hold firmly for about one minute while the machine collects the data. Afterwards, participants can continue with regular activities. For the statistical analysis of data, Microsoft Excel was used. Excel was also used to record and store the data. Subsequently, SPSS (version 26) was used to create figures and tables with a 95% confidence interval and an error margin of 0.55.

## 3. Results

The demographic makeup of the sample was 36% professors, followed by 30% students and 24% administrative staff. In terms of gender, 76% of the participants in this study were female and the remaining 24% were male. According to the results for Body Mass Index (BMI), 44% of participants were overweight, followed by 35% who were obese. Only 21% were within the normal weight range. Taking into consideration only the professors, it was found that 54% of them (more than half) were overweight, while 23% were obese and normal weight, respectively. Meanwhile, based on their BMI scores, 55% of the administrative personnel were obese, followed by 35% who were overweight and only 10% who were of normal weight. For the students, the predominant BMI category was overweight with 36% (despite the youth of this group), followed by obesity and normal weight with 32%, respectively.

**Figure 1:**
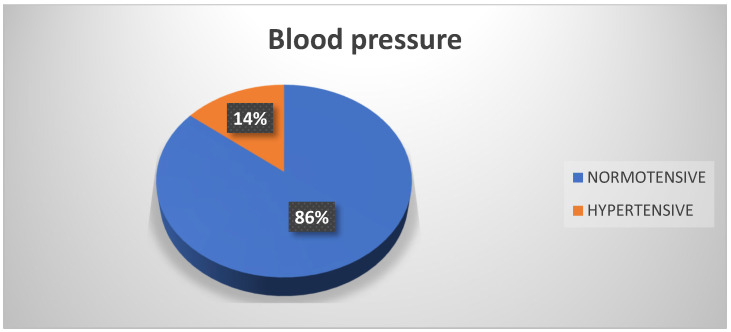
Blood pressure. Source: University community measurements.

The findings of the participant blood pressure measurements were that 86% of participants were normotensive at the time of measurement. Only 14% of participant scores indicated hypertension (Figure 1). There was no major difference in this respect according to the occupation of each participant.

**Figure 2:**
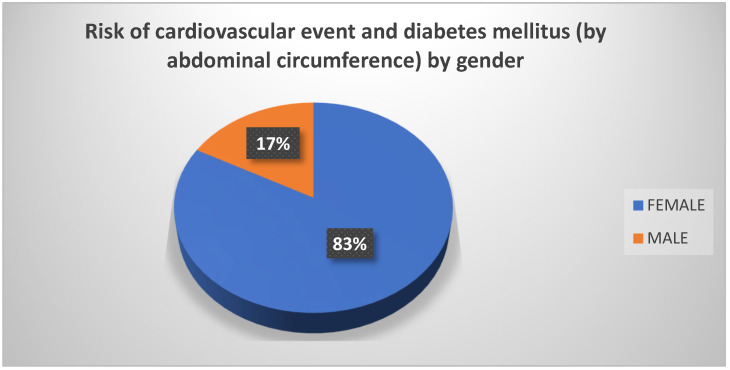
Risk of cardiovascular event and diabetes mellitus (according to abdominal circumference) by gender. Source: University community measurements.

**Figure 3:**
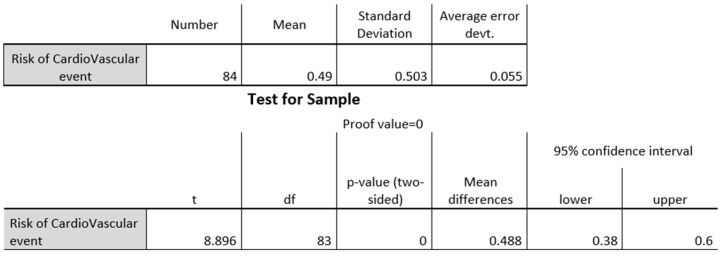

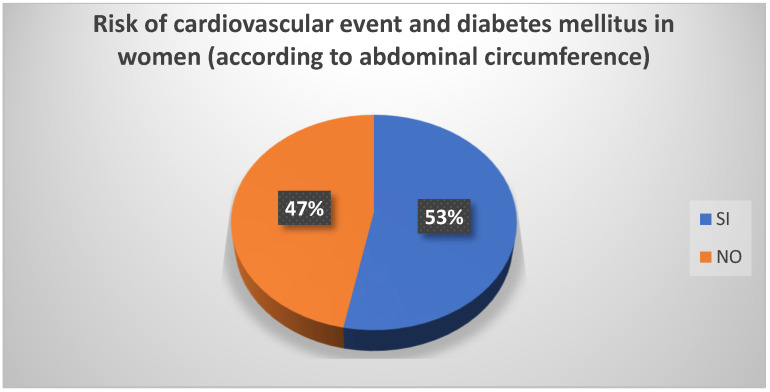
Risk of cardiovascular event and diabetes mellitus in women (according to abdominal circumference). Source: University community measurements.

When measuring the abdominal circumference of participants while taking gender into consideration, it was observed that the majority of females (83%) were at risk of experiencing a cardiovascular event or of developing diabetes mellitus later in life; only 17% of men displayed these risks (Figure 2). Additionally, within the group of women, it was found that 53% (more than half) were at risk of experiencing a cardiovascular event and of developing diabetes mellitus (Figure 3).

**Figure 4:**
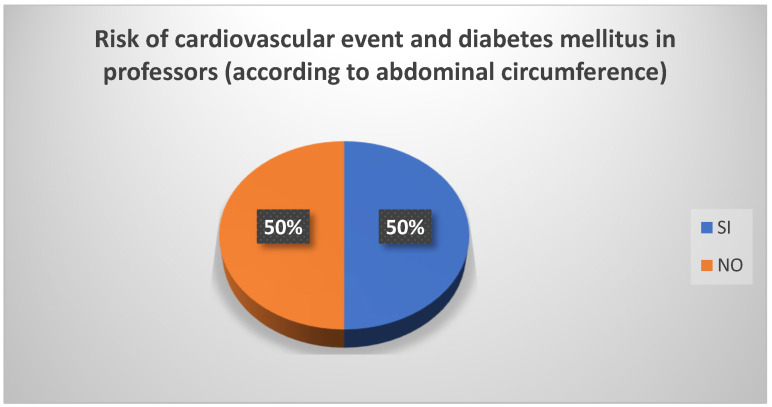
Risk of cardiovascular event and diabetes mellitus in professors (according to abdominal circumference). Source: University community measurements.

**Figure 5:**
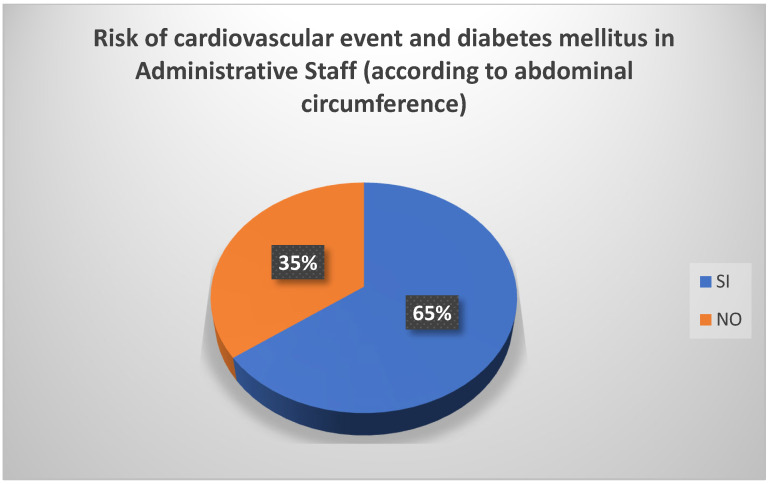
Risk of cardiovascular event and diabetes mellitus in administrative staff (according to abdominal circumference). Source: University community measurements.

Based on the occupation in a university setting, and in relation to the abdominal circumference measurement, it was found that 50% of professors were at risk of experiencing a cardiovascular event or of developing diabetes mellitus (Figure 4). The administrative staff were similarly at risk (65%) (Figure 5). Among students, the risk was minimal.

**Figure 6:**
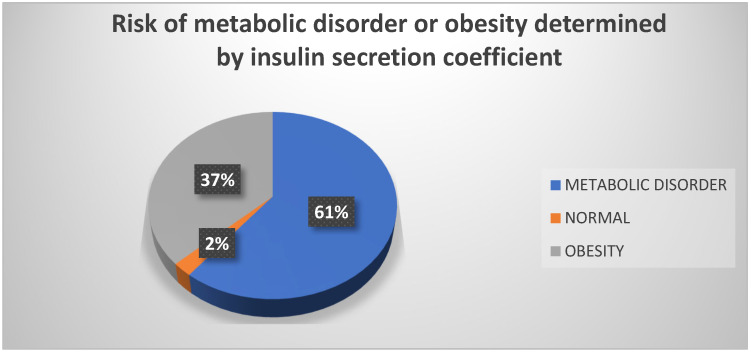
Risk of metabolic disorder or obesity, determined by insulin secretion coefficient. Source: University community measurements.

It was observed that, in terms of insulin secretion coefficient, 61% of participants were at risk of developing a metabolic disorder, followed by 37% who were at risk of becoming obese (Figure 6).

**Figure 7:**
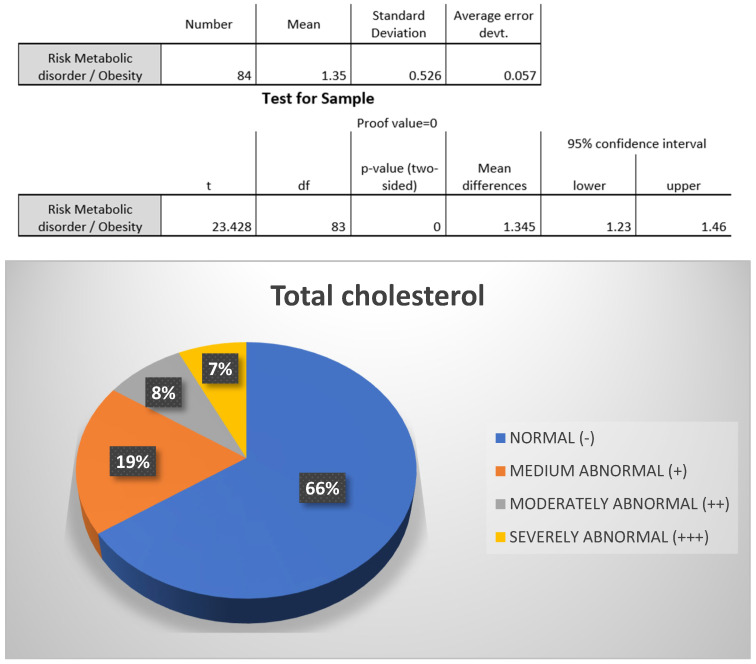
Total cholesterol. Source: University community measurements.

Regarding total cholesterol, it was observed that the majority of participants, 66%, were within the normal range, while only 19% presented with moderately abnormal total cholesterol (Figure 7).

**Figure 8:**
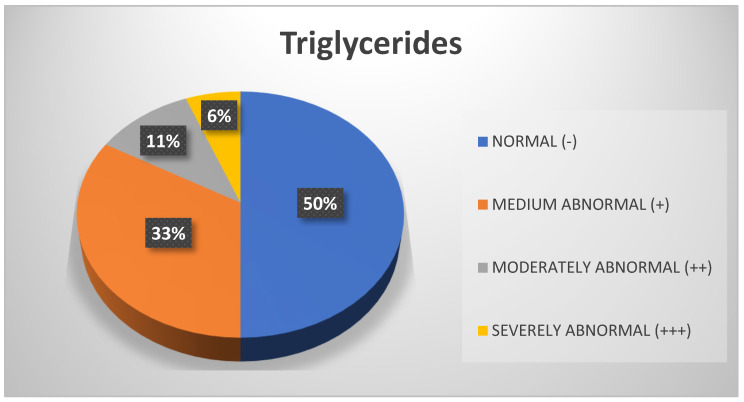
Triglycerides. Source: University community measurements.

**Figure 9:**
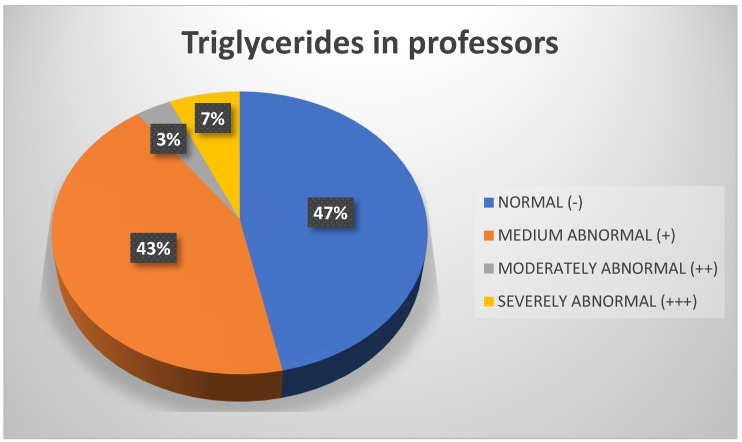
Triglycerides in professors. Source: University community measurements.

Analysis of triglycerides revealed that the values for 50% of the sample were within the normal range, followed by 33% who exhibited moderately abnormal values (Figure 8). However, considering only professors, it is notable that 43% presented with moderately abnormal values (Figure 9).

**Figure 10:**
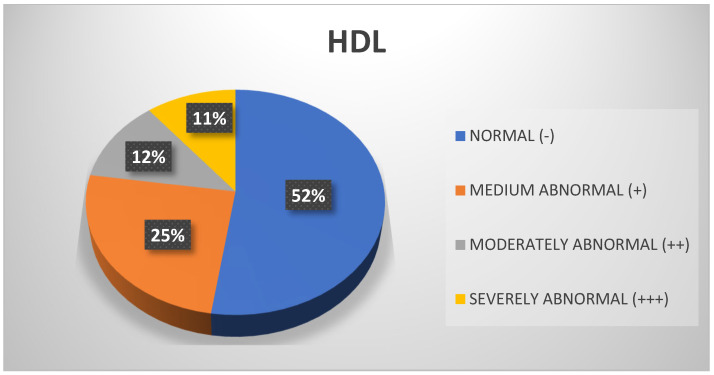
HDL. Source: University community measurements.

**Figure 11:**
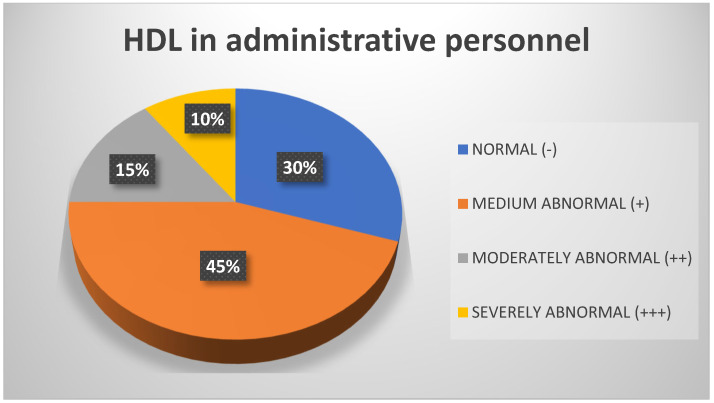
HDL in administrative personnel. Source: University community measurements.

Regarding the HDL variable, the measurements taken indicate that in general, more than half of the sample (52%) obtained values within the normal range, followed by 25% who scored moderately abnormal (Figure 10). However, regarding the administrative staff, 45% obtained moderately abnormal measurements for HDL and only 30% obtained values within the normal range (Figure 11). 

**Figure 12:**
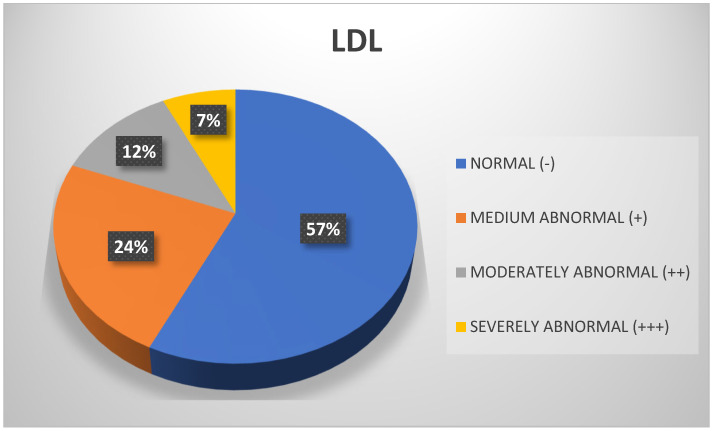
LDL. Source: University community measurements.

Regarding the LDL variable, we observed that more than half of the participants achieved values in the normal range (57%), followed by 24% with moderately abnormal scores, with no significant differences found in the particular groups by occupation (Figure 12). 

## 4. Discussion

It is of importance to understand the risk factors that predispose people to cardiovascular illnesses as the global population is increasing. In developing countries such as Ecuador, the population seems to be unaware of how regular actions and habits impact their health; thus, people tend to be non-cooperative when it comes to research. This was found to be a limitation. For this reason, sample sizes tend to be small, as it is difficult to motivate the community to participate in research studies.

In a study with a sample of 24,718 patients taken from the PURE (Prospective Urban Rural Epidemiology) system that has been used in Colombia, Brazil, Argentina and Chile, it was striking that the predominant gender presenting with cardiovascular events were men and that the area of residence associated with greater risk were rural areas. Further, the most predisposing factors were smoking, obesity, and heterogenic dyslipidemia, which is characterized by hypertriglyceridemia and increased LDL [10]. Metabolic syndrome was also assessed and two predisposing factors were found: glycemia greater than 100 mg/Dl and increased abdominal circumference. The study authors state that levels of visceral obesity should be controlled and that a reduction of about 10% of a patient’s weight is required to see improvement [10].

It is estimated that in the year 2017, 55 million people died worldwide, and of these, 17.7 million deaths were due to cardiovascular diseases (CVD). Most epidemiological studies that have linked CVD mortality with risk factors have been conducted in high-income countries (HIC), despite the fact that most CVD deaths currently occur in middle-income (MIC) and low-income (LIC) countries [11].

Recently, the morbidity and mortality results of the PURE study (Prospective Urban Rural Epidemiology), a prospective cohort population study that includes people aged 35 to 70 from 21 countries and five continents, have been published. Its primary objectives are to determine the incidence of fatal and non-fatal cardiovascular diseases, cancer, accidents, respiratory diseases, and hospital admissions. In the study, standardized incidence levels for age and sex were calculated per 1000 person-years [11].

The health sector must increase awareness and improve the diagnosis, treatment, and control of hypertension and atherogenic dyslipidemia. These are factors that can be controlled with pharmacological interventions of proven effectiveness at reducing CVD mortality, as has been demonstrated in studies such as HOPE-39-11, which included 12,500 patients, 28% of which were Ibero–American, and the HOPE-4 study, which included more than 1300 hypertensive patients [11].

A review and analysis of global epidemiological and prevention studies shows that in high-income countries, cancer has displaced cardiovascular diseases as the leading cause of mortality, unlike in middle- and low-income countries, where the leading cause of mortality is still cardiovascular diseases.

In order to take action that can help decrease the risk of cardiovascular diseases, it is recommended to involve the community in developing and implementing programs that are accessible to the population and focused on modifiable behaviors to promote habits that can have a positive impact on health, such as healthy eating, access to healthy food choices close to or on campus, as well as increasing the popularity of working out and of leading an active lifestyle.

## Data Availability

Data are contained within the article.
